# Evaluation of Anticancer Activity of Nucleoside–Nitric Oxide Photo-Donor Hybrids

**DOI:** 10.3390/molecules29143383

**Published:** 2024-07-18

**Authors:** Elena Marchesi, Elisabetta Melloni, Fabio Casciano, Elena Pozza, Roberto Argazzi, Carmela De Risi, Lorenzo Preti, Daniela Perrone, Maria Luisa Navacchia

**Affiliations:** 1 Department of Chemical, Pharmaceutical and Agricultural Sciences, University of Ferrara, 44121 Ferrara, Italy; mrclne@unife.it (E.M.); agr@unife.it (R.A.); drc@unife.it (C.D.R.); 2 Department of Translational Medicine, University of Ferrara, 44121 Ferrara, Italy; elisabetta.melloni@unife.it (E.M.); cscfba@unife.it (F.C.); pzzlne1@unife.it (E.P.); 3 LTTA Centre, University of Ferrara, 44121 Ferrara, Italy; 4 Institute for Organic Synthesis and Photoreactivity (ISOF), National Research Council of Italy (CNR), 40129 Bologna, Italy; 5 Department of Environmental and Prevention Sciences, University of Ferrara, 44121 Ferrara, Italy; prtlnz@unife.it

**Keywords:** nucleoside analogs, hybrid drugs, nitric oxide, photo-donor, anticancer activity, multitarget anticancer drugs

## Abstract

Herein, we report the synthesis of a new hybrid compound based on a 2′-deoxyuridine nucleoside conjugated with a NO photo-donor moiety (dU-t-NO) via CuAAC click chemistry. Hybrid dU-t-NO, as well as two previously reported 2′-deoxyadenosine based hybrids (dAdo-S-NO and dAdo-t-NO), were evaluated for their cytotoxic and cytostatic activities in selected cancer cell lines. dAdo-S-NO and dAdo-t-NO hybrids displayed higher activity with respect to dU-t-NO. All hybrids showed effective release of NO in the micromolar range. The photochemical behavior of the newly reported hybrid, dU-t-NO, was studied in the RKO colon carcinoma cell line, whereas the dAdo-t-NO hybrid was tested in both colon carcinoma RKO and hepatocarcinoma Hep 3B2.1-7 cell lines to evaluate the potential effect of NO released upon irradiation on cell viability. A customized irradiation apparatus for in vitro experiments was also designed.

## 1. Introduction

Cancer is a multifactorial disease resulting from interactions of complex genetic and environmental factors and, nowadays, is one of the most deadly diseases threatening the health of populations globally [1]. Colorectal cancer is one of the most common malignancies worldwide and can be recorded as the second leading cause of cancer-associated deaths in women and the third most common disease in men [2]. According to a recent estimation by the International Agency for Research on Cancer, liver cancer is the sixth most frequently diagnosed cancer and the fourth leading cause of cancer death for both sexes globally [3]. Indeed, more than 900 k people were diagnosed with liver cancer and almost 800k died of liver cancer worldwide in 2020 [4]. Surgical approaches are the best option for both colorectal and liver cancer treatment; however, for malignancies diagnosed at advanced stages, systemic approaches including targeted therapy, immunotherapy, and chemotherapy are necessary. Unfortunately, a significant fraction of cancers show resistance to conventional chemotherapeutic agents [5]. In the last decades, significant efforts have been directed towards the development of combination therapies, which involve the use of different chemodrugs with diverse molecular mechanisms that can act cooperatively and prevent cancer cells from developing resistance mechanisms [6,7,8]. However, the advantages of administering multiple agents are often limited by dose-limiting toxicities and drug–drug interactions [7]. Therefore, the design of multitarget anticancer agents able to simultaneously interfere with different enzymes or signaling pathways of cancer progression is an urgent need. 

Molecular hybridization is a relatively new strategy in drug discovery aimed at designing new drugs by combining two or more pharmacophore units through covalent bonds [9,10]. Therefore, hybrid drugs can provide combination therapies by exploiting a single multi-functional unit, leading to a pharmacological effectiveness greater than the sum of each individual component’s efficacy [11,12,13]. Over the last decades, the design and synthesis of hybrid compounds as multitarget anticancer agents has been a research field in great expansion [10,14]. 

Nitric oxide (NO), besides its well-known role in the bioregulation of physiological processes [15], has emerged as an interesting molecule for cancer treatment [16,17,18]. NO’s biological effects on cancer are markedly dependent on its concentration. While low levels of NO (in the pM to nM range) can exert pro-tumor effects such as angiogenesis, enhanced cell proliferation, migration, survival, invasiveness, and metastasis, high levels of NO (in the μM range) exhibit a pro-oxidant cytotoxic effect by inducing cell apoptosis, inhibition of angiogenesis, and mitochondrial respiration, as well as by sensitizing tumors to therapeutics [19,20,21,22,23]. Due to its short lifetime (ca. 5 s in tissues) [24,25], NO’s region of action is confined to a short distance from its production site inside the cell, thus reducing systemic toxicity effects and improving treatment effectiveness. However, the critical dependence of NO’s biological effects on concentration, as well as its high reactivity, allows only a limited usage of gaseous NO by direct inhalation in clinical medicine [26,27]. Thus, extensive studies on the design of molecular and supramolecular NO donors for efficient NO delivery to specific disease lesions have received much attention [28,29,30,31]. Among others, small molecules able to release NO under a specific external stimulus, for instance, photoactivated NO donors, have been considered quite attractive due to the possibility of precisely controlling NO production and NO release at the target site [32].

**Figure 1:**
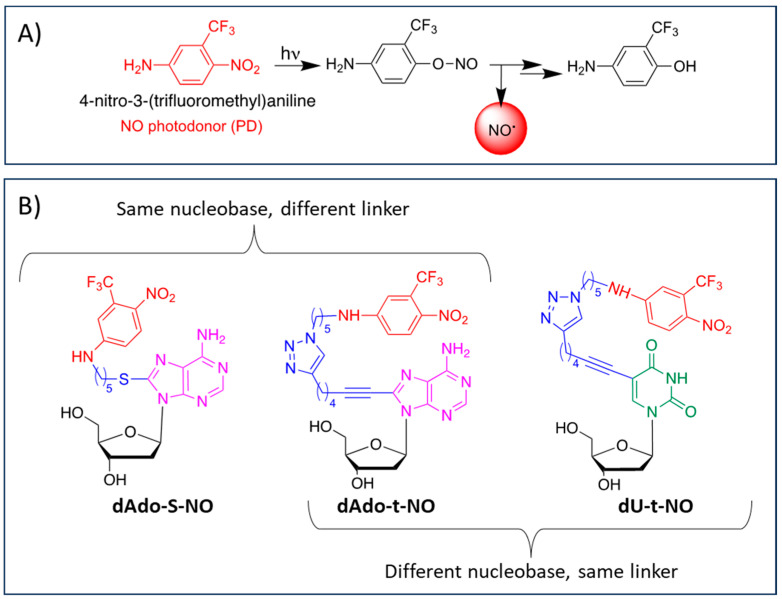
(**A**): molecular structure of 4-nitro-3-(trifluoromethyl)aniline and mechanism of NO release; (**B**) molecular structure of hybrids dAdo-S-NO [35], dAdo-t-NO [37], and dU-t-NO.

Our interest in the study of multitarget hybrid compounds for pharmacological applications has prompted us to explore hybrids integrating a NO donor molecule in response to specific stimuli, such as 4-nitro-3-(trifluoromethyl)aniline, which is able to release NO upon irradiation through the mechanism depicted in Figure 1A [33]. The nitrobenzene unit of 4-nitro-3-(trifluoromethyl)aniline is not toxic to the cells before and after the release of NO [34], and the presence of a free NH_2_ group, a chemical reactive moiety, can serve as a conjugation point without interfering with light-triggering [33].

In the framework of a study on the conjugation of either nucleosides or bile acids with 4-nitro-3-(trifluoromethyl)aniline, we reported, in a previous paper [35], the photophysical properties and the in vitro antiproliferative activity of a small library of hybrids presenting, as conjugation partners, a nucleoside molecule such as 2′-deoxyadenosine or selected bile acids such as urso- and cheno-deoxycholic acids in leukemic K562 and colon carcinoma HCT-116 cell lines.

Photochemical experiments demonstrated the effective release of NO from all hybrids under the exclusive control of visible light inputs. Moreover, in the case of hybrid dAdo-S-NO (Figure 1B), the antiproliferative activity against HCT-116 cells was tested both in the dark and upon illumination. After 40 min light irradiation (λ= 420 nm, 7 mW cm^−2^) the IC_50_ value of the hybrid dAdo-S-NO was more than halved, decreasing from 66.2 µM to 31.0 µM [35]. This result, being the first evidence of in vitro combined chemo- and photo-therapeutic activities for the same molecule, prompted us to design some more nucleoside–PD hybrids by changing the nucleobase and/or the linker unit (Figure 1B). 

Nucleosides are small endogenous bioactive molecules characterized by different chemical/biochemical reactive moieties. Therefore, they represent an interesting platform for conjugation with functional molecules. From a biological point of view, several nucleosides can behave as antimetabolites and can inhibit cellular division and viral replication by incorporation into DNA or RNA, resulting in potential therapeutic benefits. Numerous nucleoside analogs have been developed as anticancer drugs over the past decades [36].

**Scheme 1:**
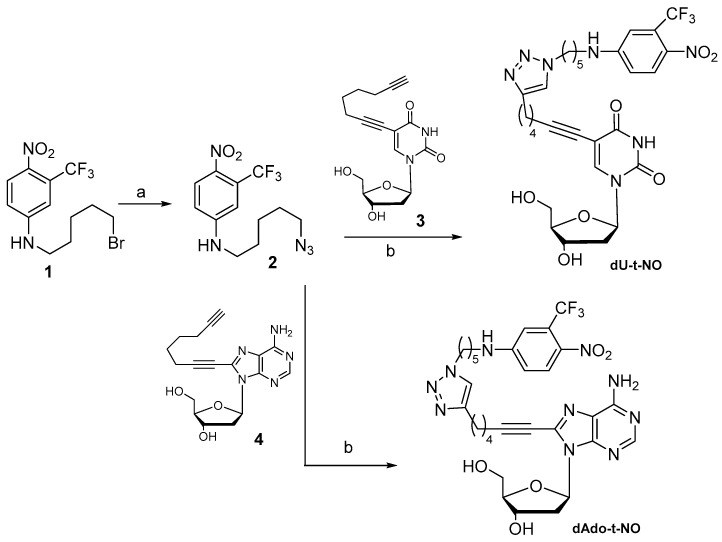
Synthesis of hybrids dU-t-NO and dAdo-t-NO. (a) NaN_3_, DMF, 65 °C, 4 h (91% yield); (b) CuSO_4_·5H_2_O, sodium ascorbate, THF/t-BuOH/H_2_O (1.5:1:1), microwaves: 80 °C, 30 min (65−68% yield).

Herein, we reported the synthesis and the chemical and biological characterization of a novel 2′-deoxyuridine-based hybrid (namely dU-t-NO, Figure 1B) obtained by conjugation via copper-catalyzed azide-alkyne cycloaddition (CuAAC) click chemistry of *N*-(5-azidopentyl)-4-nitro-3-(trifluoromethyl)aniline **2** with 2′-deoxyuridine alkyne derivative **3** (Scheme 1). In addition, two hybrids based on 2′-deoxyadenosine that were previously reported by some of us, i.e., dAdo-S-NO [35] and dAdo-t-NO [37] (Figure 1B), conjugated with the 4-nitro-3-(trifluoromethyl)aniline PD unit through different linkers such as a thioalkyl chain and a triazole ring, respectively, were considered. All hybrids underwent photophysical characterization, cytotoxicity investigation, and cell cycle analysis in human cells of different histopathological origin, such as RKO colon carcinoma and Hep 3B2.1-7 hepatocarcinoma cell lines. The effect of NO release on cell viability was also evaluated in RKO cancer cells for the dU-t-NO hybrid and in both RKO and Hep 3B2.1-7 cell lines for the dAdo-t-NO hybrid.

## 2. Results

### 2.1. Chemistry

#### 2.1.1. Synthesis of Nucleoside–NO Photo-Donor Hybrids

The synthesis of the dU-t-NO and dAdo-t-NO hybrids is shown in Scheme 1. Although compound dAdo-t-NO has been previously investigated for its biological activity against several cellular lines [37], we report here, for the first time, its synthesis and its chemical characterization. Alkynes **3** and **4** [38,39] and bromo derivative **1** [35] were prepared as previously reported by some of us, whereas *N*-(5-azidopentyl)-4-nitro-3-(trifluoromethyl)aniline building block **2** was prepared from the corresponding bromo derivative **1** by reaction with NaN_3_ in an almost quantitative yield (Scheme 1). Azide derivative **2** was reacted with alkynes **3** and **4** via CuAAC under microwave conditions to obtain the corresponding pure hybrids dU-t-NO and dAdo-t-NO with good yields after chromatographic purification (Scheme 1). Hybrid dAdo-S-NO was prepared as previously reported by our group [35].

#### 2.1.2. Chemical Stability in Cell Culture Medium

The chemical stability of all hybrids in cell culture medium (DMEM) at 37 °C was established by means of HPLC-UV-MS analyses. A time course at 3, 6, and 14 h demonstrated that the hybrids were stable up to 14 h. HPLC-UV-MS studies allowed us to detect, in all cases, only trace amounts of the corresponding products derived from hydrolysis of the *N*-glycosidic bond, as they were the only degradation products after up to 14 h in a cell culture medium at 37 °C.

### 2.2. Photochemistry

**Figure 2:**
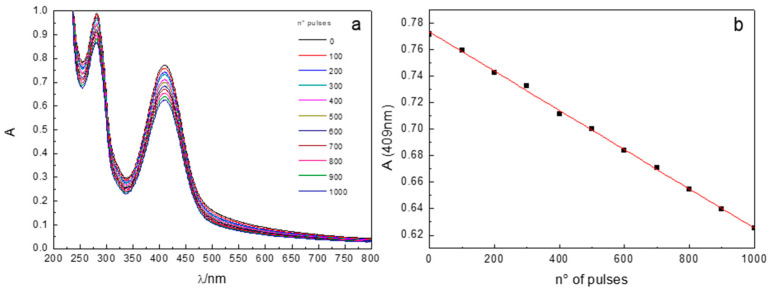
(**a**): Absorbance changes of a dAdo-S-NO 50 μM water solution irradiated with 355 nm laser pulses; (**b**): linear fit of the absorbance drop at peak wavelength (409 nm).

In order to quantify the NO photorelease efficiency of the PD unit under specific concentration and irradiation conditions, the photochemical reaction quantum yield for all three hybrids was determined in a water solution. A pulsed laser source, with an emission wavelength lying within the electronic absorption band of the PD unit, was chosen to precisely control the number of photons delivered to the sample in each shot set. In a typical experiment, a volume of 3 mL of a 50 μM water solution of each hybrid, placed in a fluorimetric fused silica cuvette, was irradiated at 1 Hz with laser pulses at 355 nm with pulse energies in the range of 3–5 mJ/pulse. In all cases, zero-order kinetics was found to lead to a linear absorbance drop within a 20% total absorbance change, as shown in Figure 2 for dAdo-S-NO. The number of absorbed photons was calculated from the measured pulse energy and corrected for the 355 nm absorbance change.

**Figure 3:**
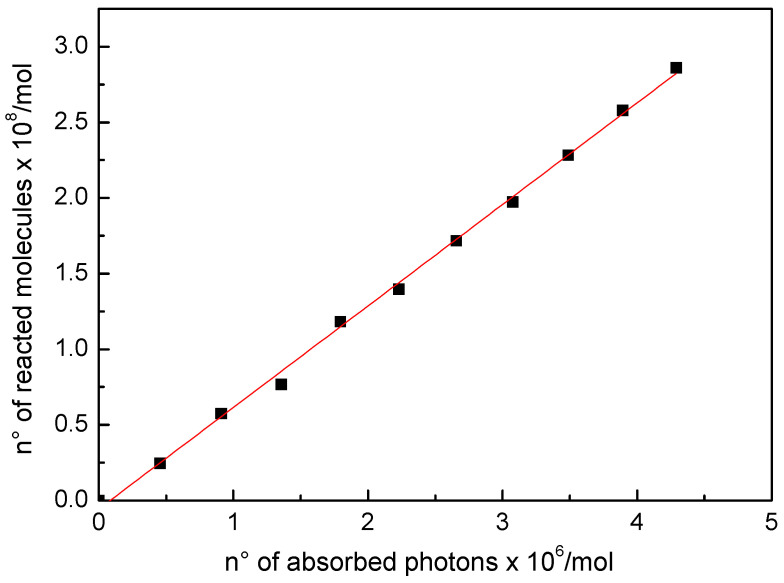
Number of reacted molecules vs. number of absorbed photons for dAdo-S-NO in water. Quantum yield values were found to be (6.7 ± 0.1) × 10^−3^, (4.6 ± 0.2) × 10^−3^, and (5.95 ± 0.08) × 10^−3^ for dAdo-S-NO, dAdo-t-NO, and dU-t-NO, respectively.

The photoreaction quantum yield was obtained from the slope of the straight line resulting from the plot of the number of reacted molecules vs. the number of absorbed photons (Figure 3).

In a preliminary set of experiments, a 380 nm LED matrix was used to irradiate the cell cultures at radiant power densities ranging from 1 to 5 mW/cm^2^ and for decreasing times, starting from 4 h until 10 min. This particular peak wavelength was initially chosen to reach an optimum spectral match with the absorption band of the PD unit, with the aim of maximizing the photogeneration of NO. In all cases, the cells did not survive the treatment, showing that the wavelengths were too short to irradiate them within a safe limit. This result suggested the use of longer wavelengths and lower power densities, keeping in mind the necessity of photogenerating a sufficient amount of NO to observe an effect on cells upon irradiation. Subsequent experiments performed with a single LED source at a peak of 420 nm showed that wavelengths shorter than 410 nm were still detrimental and filtering them off was a necessity. With the use of a 420 nm cut-off filter, the LED spectral profile was finally cleaned, leading to a cell-safe irradiating time of 40 min, which was maintained for all in vitro tests.

**Figure 4:**
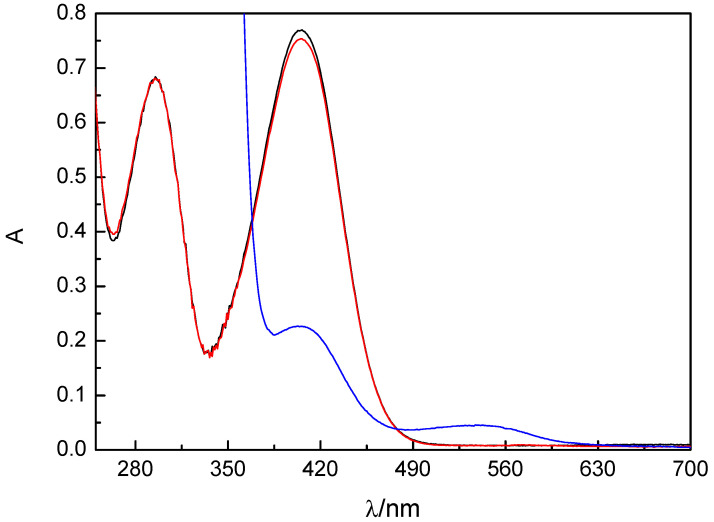
Absorption change upon 40 min irradiation of a 50 μM aqueous solution of dU-t-NO with a 420 nm LED source at 1 mW/cm^2^. The spectrum after the addition of the Griess reagent is shown (blue line).

The actual photogeneration of NO in a water solution was checked by evaluating, through the Griess test, the amount of nitrite formed upon irradiation of the hybrids for 40 min with a 420 nm LED source. A volume of 3 mL of a 50 μM solution of each hybrid, in a 1 cm path length cuvette, was uniformly irradiated with the apparatus described in Materials and Methods (see Section 4.2.2.), held horizontally such as to have a radiant power density of 1 mW/cm^2^ on the entrance window of the cuvette. After irradiation, 1 mL of the solution was added to an equal volume of sulfanylamide (Promega) and kept for 5 min in the dark. A 1 mL amount of NED (Promega) was then added, and the resulting solution was placed in the dark for a further 5 min. The absorption spectrum recorded showed the typical absorption band of the azo compound, with a maximum at 540 nm (Figure 4). A calibration curve was obtained using standard solutions of NaNO_2_ in water in the concentration range of 2–20 μM. Nitrite concentrations lower than 1 μM were measurable.

All three hybrids produced results similar to those reported in Figure 4 for dU-t-NO, showing the capability to generate a NO concentration of about 2 μM from a 50 μM hybrid solution in the same irradiation time used for experiments on cells.

### 2.3. Biological Evaluation 

#### 2.3.1. Evaluation of the Biological Activity of dAdo-S-NO, dAdo-t-NO, and dU-t-NO on Colon and Hepatocarcinoma Cancer Cells in the Dark

**Figure 5:**
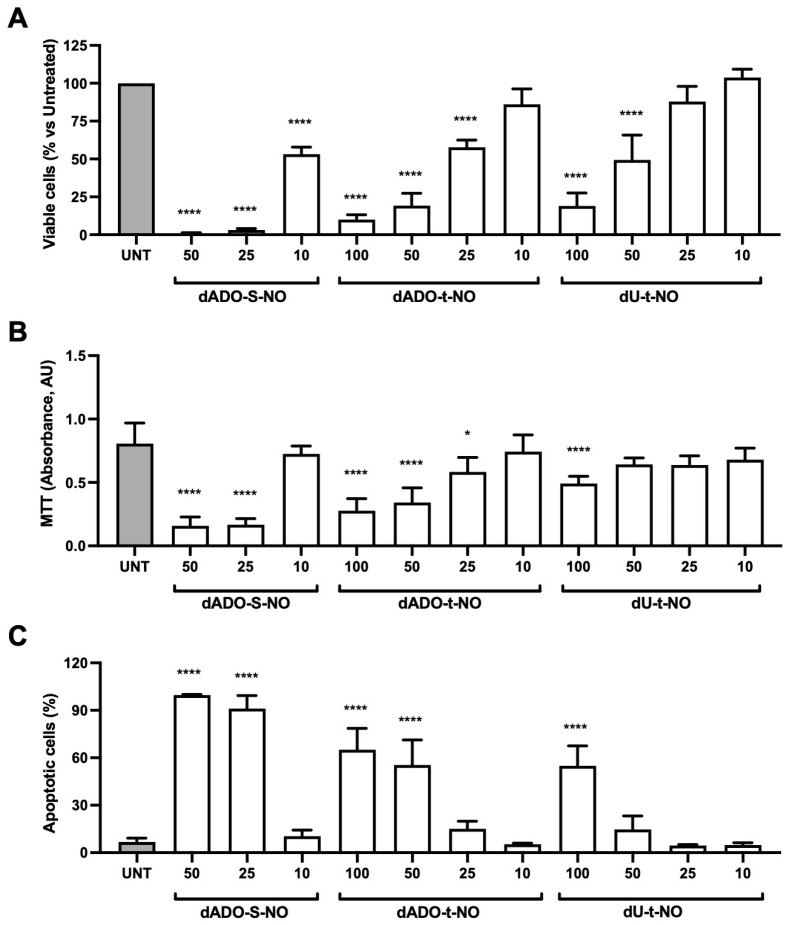
Biological effects of dAdo-S-NO, dAdo-t-NO, and dU-t-NO on the RKO cell line. Cell viability (**A**), proliferation (**B**), and apoptosis (**C**) evaluation in RKO cells treated for 72 h with three different dAdo-S-NO concentrations (50; 25; 10 μM) or with four different concentrations (100; 50; 25; 10 μM) of each of the following hybrids: dAdo-t-NO or dU-t-NO. Incubations with the hybrids were performed in the dark. Results are reported as mean ± SD of at least three independent experiments. Statistical analysis was performed using one-way ANOVA followed by the Bonferroni post-hoc test. * *p* < 0.05, **** *p* < 0.0001 with respect to untreated cells (UNT).

The biological effects of the three hybrids in the absence of irradiation were firstly assayed on the RKO colon carcinoma cell line. As shown in Figure 5A,B, dAdo-S-NO, dAdo-t-NO, and dU-t-NO induced a dose-dependent reduction in the number and proliferation of RKO viable cells after 72 h of treatment, as demonstrated by the Trypan Blue dye exclusion test and the MTT assay. 

In particular, dAdo-S-NO was shown to cause a dose-dependent reduction in the number and proliferation of RKO viable cells at all the tested concentrations, especially when used at the concentrations of 25 and 50 μM. On the other hand, dAdo-t-NO was significantly effective in the range of 25–100 μM; however, it induced only a slight reduction in cell viability when used at the concentration of 10 μM. Conversely, the dU-t-NO hybrid was able to significantly reduce the number of viable cells at 50 and 100 μM and only slightly at 25 μM, but it seemed to be almost ineffective if used at 10 μM. The evaluation of the apoptosis levels induced in the RKO cell line by dAdo-S-NO, dAdo-t-NO, and dU-t-NO essentially reflected the dose-dependent effects exerted on cell viability. Indeed, a significant apoptotic effect was achieved by dAdo-S-NO at 25 and 50 μM, dAdo-t-NO at 50–100 μM, and dU-t-NO at 100 μM (Figure 5C).

**Figure 6:**
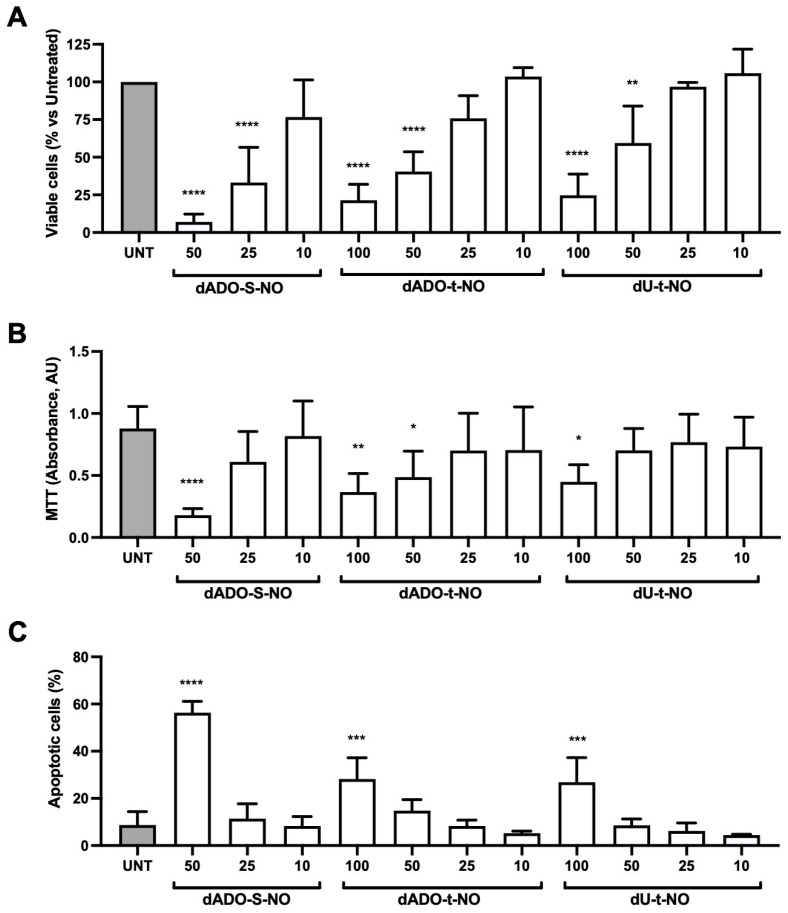
Biological effects of dAdo-S-NO, dAdo-t-NO, and dU-t-NO on the Hep 3B2.1-7 cell line. Cell viability (**A**), proliferation (**B**), and apoptosis (**C**) evaluation in Hep 3B2.1-7 cells treated for 72 h with three different dAdo-S-NO concentrations (50; 25; 10 μM) or with four different concentrations (100; 50; 25; 10 μM) of each of the hybrids: dAdo-t-NO or dU-t-NO. Incubations with the hybrids were performed in the dark. Results are reported as mean ± SD of at least three independent experiments. Statistical analysis was performed using one-way ANOVA followed by the Bonferroni post-hoc test. * *p* < 0.05, ** *p* < 0.01, *** *p* < 0.001, **** *p* < 0.0001 with respect to untreated cells (UNT).

Analogously, the analysis of the effect of the hybrids on the Hep 3B2.1-7 hepatocarcinoma cell line in the dark demonstrated their effectiveness in reducing cell viability in a dose-dependent manner (Figure 6). The most important effects on this cell line in terms of reductions in cell number and proliferation were shown by dAdo-S-NO at the concentrations of 25 and 50 μM and by dAdo-t-NO and dU-t-NO at 50–100 μM, even if dU-t-NO was a little less effective when compared to dAdo-t-NO (Figure 6A,B). 

The differences in the effectiveness of the three hybrids on RKO and Hep 3B2.1-7 cells could also be appreciated by observing their IC_50_ values, shown in Table 1, which also reports the IC_50_ values for Paclitaxel (used as a reference drug). 

In the case of Hep 3B2.1-7 cells, a significant but noteworthy rate of apoptosis was induced only by higher concentrations of the three hybrids, as shown in Figure 6C. Of note, we chose to use different ranges of concentrations for dAdo-S-NO (50–10 μM) and dAdo-t-NO/dU-t-NO (100–10 μM) on the basis of the differing effectiveness on both the RKO and Hep 3B2.1-7 cell lines.

**Figure 7:**
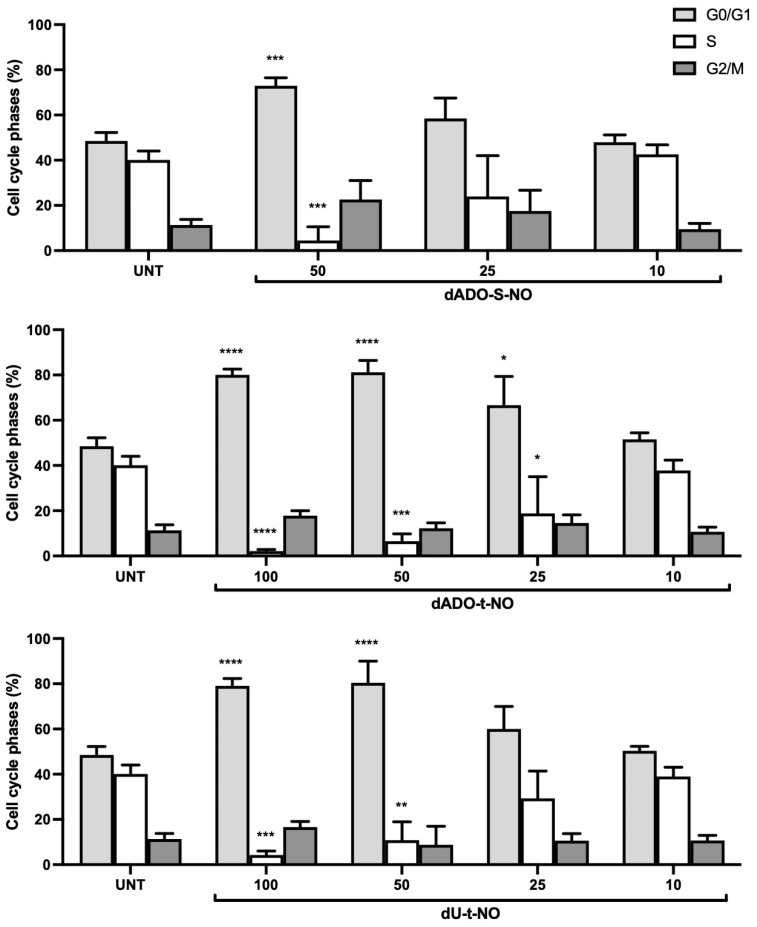
Effects of the three hybrids on the cell cycle of RKO cells. Cells’ distribution in the cell cycle phases after treatment for 36 h with three different dAdo-S-NO concentrations (50; 25; 10 μM) or with four different concentrations (100; 50; 25; 10 μM) of each of the hybrids, dAdo-t-NO or dU-t-NO, is shown. Incubations with the hybrids were performed in the dark. Results are reported as mean ± SD of at least three independent experiments. Statistical analysis was performed by one-way ANOVA followed by the Bonferroni post-hoc test. * *p* < 0.05, ** *p* < 0.01, *** *p* < 0.001, **** *p* < 0.0001 with respect to untreated cells (UNT).

**Figure 8:**
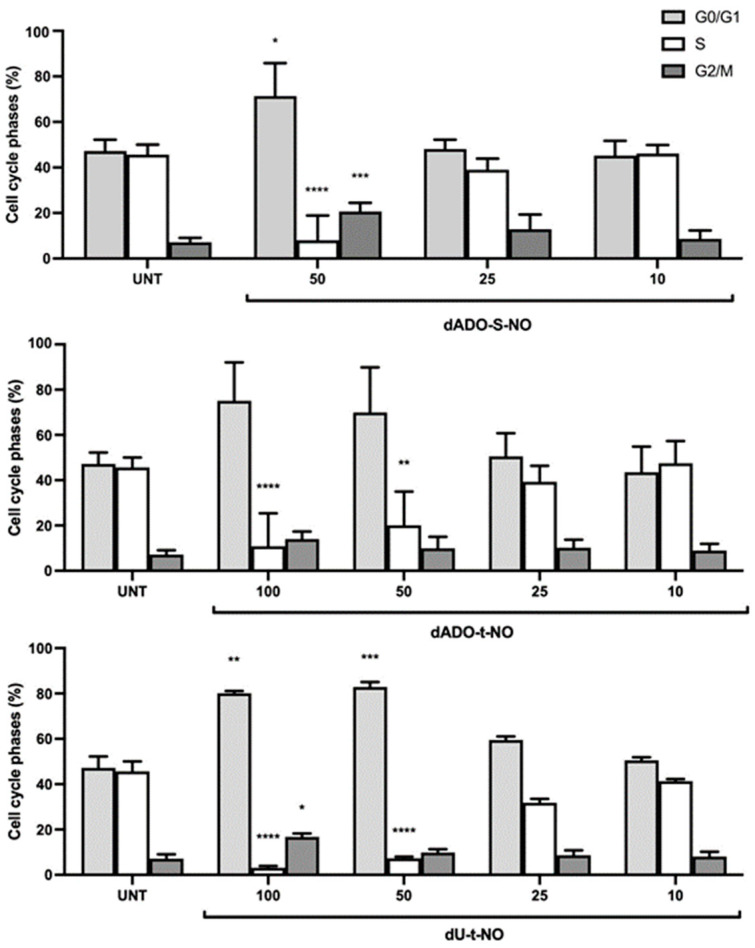
Effects of the three hybrids on the cell cycle of Hep 3B2.1-7cells. Cells’ distribution in the cell cycle phases after treatment for 36 h with three different dAdo-S-NO concentrations (50; 25; 10 μM) or with four different concentrations (100; 50; 25; 10 μM) of each of the hybrids (dAdo-t-NO or dU-t-NO) is shown. Incubations with the hybrids were performed in the dark. Results are reported as mean ± SD of at least three independent experiments. Statistical analysis was performed by one-way ANOVA followed by the Bonferroni post-hoc test. * *p* < 0.05, ** *p* < 0.01, *** *p* < 0.001, **** *p* < 0.0001 with respect to untreated cells (UNT).

In these first sets of experiments, performed without cell irradiation, we also analyzed the effect of the hybrids on the cell cycle of RKO and Hep 3B2.1-7 cells. As shown in Figure 7 and Figure 8, all three compounds caused an arrest of the cell cycle in a dose-dependent manner, an effect that was nearly ineffective at the concentration of 10 μM. Of note, a concentration of 25 μM was demonstrated to be less effective on Hep 3B2.1-7 cells than on the RKO cell line.

#### 2.3.2. Evaluation of the Biological Activity of dU-t-NO on RKO Colon Carcinoma and dAdo-t-NO on RKO Colon Carcinoma and Hep3B2.1-7 Hepatocarcinoma Cell Lines after Irradiation

**Figure 9:**
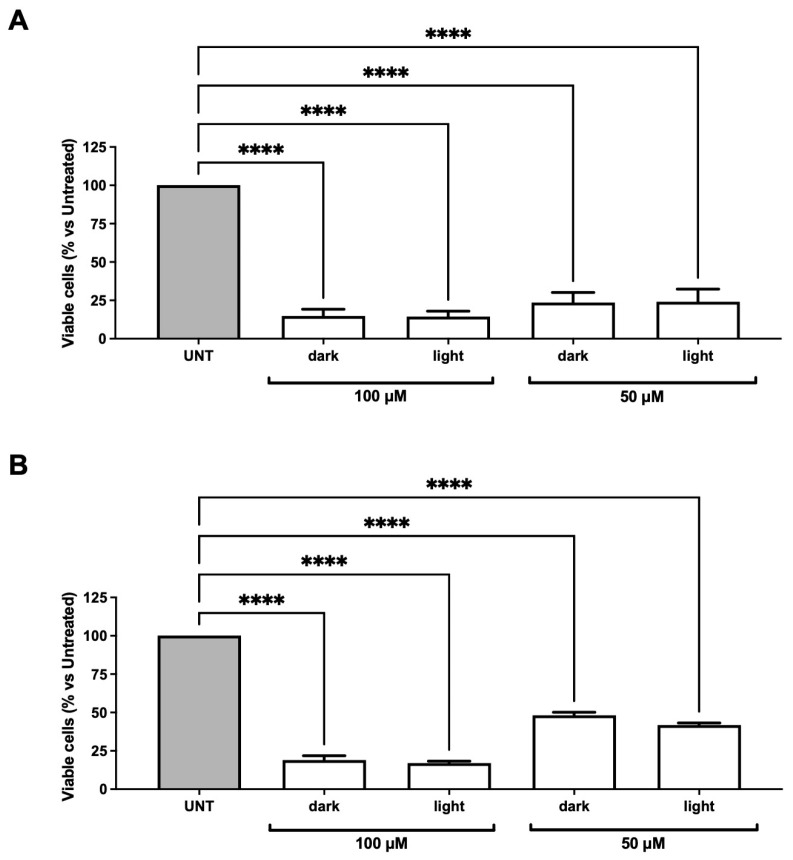
Comparative effects of dAdo-t-NO and dU-t-NO on reductions in RKO cell number in the dark or after irradiation (light). The effects of 50 and 100 μM of dAdo-t-NO (**A**) and dU-t-NO (**B**) hybrids are shown. Results are reported as mean ± SD of at least three independent experiments. Statistical analysis was performed by one-way ANOVA followed by the Bonferroni post-hoc test. **** *p* < 0.0001.

With the aim of generating NO levels able to exert anticancer activity through photochemical release, we chose to test dAdo-t-NO and dU-t-NO at the highest concentrations (50 and 100 μM) in RKO cells [20]. The analysis of the effects of dAdo-t-NO on viable cell number showed that no significant difference could be detected between experiments performed in the dark and those in which cells were irradiated accordingly with the experimental design described in the Material and Methods section (Figure 9A). No difference could be detected also in the analysis of apoptosis when cells were treated in the dark or upon illumination. Similarly, the effect on the percentage of viable RKO cells induced by dU-t-NO in the absence or presence of light showed no statistical difference (Figure 9B), which was also the case for the induction of apoptosis. Of note, no significant difference has been observed for both hybrids in terms of basal (untreated cells) viability and apoptosis rate when comparing untreated with irradiated cells after the total incubation time (72 h), excluding the fact that irradiation could itself cause a basal reduction in cell viability.

**Figure 10:**
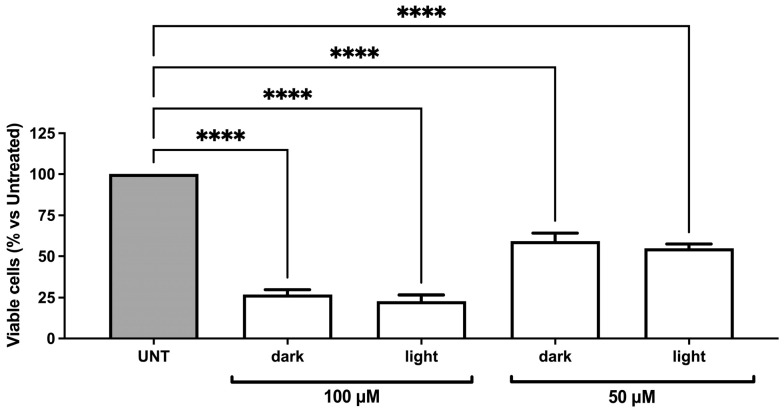
Effects of dAdo-t-NO on decreases in Hep3B2.1-7 cell numbers in the dark or after irradiation (light). The effects of 50 and 100 μM of the dAdo-t-NO hybrid are shown. Results are reported as mean ± SD of at least three independent experiments. Statistical analysis was performed by one-way ANOVA followed by the Bonferroni post-hoc test. **** *p* < 0.0001.

The d-Ado-t-NO hybrid was also tested upon irradiation in the Hep3B2.1-7 hepatocarcinoma cell line. The analysis of the effects of dAdo-t-NO on viable cell numbers showed no significant difference between experiments performed in the dark and after irradiation (Figure 10). Moreover, no difference could be detected regarding the induction of apoptosis when cells were treated in the dark or upon illumination. Of note, also in the case of Hep 3B2.1-7 cells, as seen for the RKO cell line, no differences could be revealed between the percentage of viable cells or the apoptosis rate of untreated or irradiated cells after the overall time of the experiments at basal conditions (untreated cells).

## 3. Discussion

In the case of the hybrids dU-t-NO and dAdo-t-NO, the conjugation at the C-5 and C-8 positions of 2′-deoxyuridine and 2′-deoxyadenosine (Figure 1B), respectively, was achieved through CuAAC (click chemistry). The 1,2,3-triazole ring, resulting from the reaction of a terminal carbon–carbon triple bond with an azido group, can serve as both a bioisostere and a linker. The triazole ring has been known to improve the pharmacological profile of bioactive molecules thanks to its stability under physiological and enzymatic conditions; thus, it can be considered a pharmacophore itself [40]. Click chemistry has been recognized as a powerful tool for developing new structural motifs and it has been extensively employed in the field of drug discovery [37,38,39,41]. Click chemistry has also been recognized as an environmentally friendly reaction thanks to the formation of a single product in high yield, favorable atom economy, elimination of byproducts, water compatibility, and use of simple purification processes [42]. 

On the other hand, the dAdo-S-NO hybrid was prepared in a straightforward manner by nucleophilic substitution at the C-8 position of 2′-deoxyadenosine through a reaction in water [35]. 

Of note, the substitution at the C-5 and C-8 positions of pyrimidine and purine nucleobases, respectively, allows us to preserve the intrinsic recognition characteristics of natural nucleic acids through specific hydrogen bond patterns such as Watson–Crick and Hoogsteen interactions. 

dAdo-S-NO and dAdo-t-NO hybrids, both containing the 2′-deoxyadenosine moiety, differ in the nature and length of their linkers: in the case of dAdo-S-NO, the nucleoside and the PD unit are covalently linked through an S-alkyl chain, whereas, in the case of dAdo-t-NO, a 1,2,3-triazole ring was introduced (Figure 1B). A comparison of the antiproliferative activity of the two hybrids showed that dAdo-S-NO is ca. 2.5-fold more potent than dAdo-t-NO in both RKO and Hep 3B2.1-7 cell lines (Table 1). 

The dAdo-t-NO and dU-t-NO hybrids are characterized by the presence of 1,2,3-triazole and an alkyl spacer of the same length; however, they differ in the nature of the nucleobase. The data obtained for the antiproliferative activity indicated that the dAdo-t-NO hybrid, containing the purine nucleobase 2′-deoxyadenosine, is 1.9- and 1.4-fold more active in the RKO and Hep3B2.1-7 cell lines, respectively, than the dU-t-NO hybrid, which is characterized by a pyrimidine 2′-deoxyuridine unit (Table 1). 

Overall, the dAdo-S-NO hybrid was the most active of the series, being ca. 2.5-fold more potent than the dAdo-t-NO hybrid in both cancer cell lines tested and 5- and 3.5-fold more active than the dU-t-NO hybrid in the RKO and Hep3B2.1-7 cell lines, respectively (Table 1). 

Both hybrids, dAdo-S-NO and dAdo-t-NO, displayed some cytoselectivity towards RKO cells with respect to Hep3B2.1-7 cells (SI = 1.7 and 1.6, respectively), whereas the dU-t-NO hybrid showed similar antiproliferative/cytotoxic effects towards both cancer cell lines tested (Table 1).

Mechanistic studies indicated that all hybrids considered induced G0/G1 cell cycle arrest in RKO and Hep3B2.1-7 cell lines. In particular, dAdo-S-NO and dAdo-t-NO showed a more marked effect in RKO cells with respect to the Hep3B2.1-7 cell line at a concentration of 25 μM, whereas dU-t-NO showed a similar effect in both cancer cell lines. This may partly account for the cytoselectivity observed in the case of dAdo-S-NO and dAdo-t-NO towards the RKO cell line. 

Accordingly, all hybrids tested showed a significant apoptotic effect in RKO cells, whereas apoptosis was found less markedly in Hep 3B2.1-7 cells. These data reinforce the idea of the hybrids’ cytoselective activity, which seems to act not only on cell cycle arrest but also on their cytotoxic effect.

The photochemical behavior of the newly reported hybrid dU-t-NO and of the 2′-deoxyadenosine-based dAdo-t-NO hybrid was also studied in the RKO cell line in order to evaluate if NO released upon irradiation could affect cell viability. 

Cells were irradiated following the protocol reported in Materials and Methods (see Section 4.3.3), assuring safe conditions for living cells. Indeed, prolonged irradiation time (over 40 min) led to significant cell death that was not evaluable immediately after irradiation but became clear after observing cells during the following hours of incubation until the 72 h established for our experimental design. 

On the basis of NO’s concentration-dependent impact on anticancer effects [20] and the above-reported photochemical experiments that showed a release of NO of about 2 μM (from a 50 μM hybrid concentration), both hybrids were tested in vitro at 50 and 100 μM concentrations upon irradiation. Results showed no differences in the percentage of RKO viable cells treated with both dU-t-NO and dAdo-t-NO in the presence of light with respect to cells treated and kept in the dark at both concentrations. Analogous results were obtained in Hep3B2.1-7 cells treated with dAdo-t-NO under the same conditions. Accordingly, light did not improve apoptosis induction with respect to the treatment performed in the dark. These results showed that the NO released did not play any additive role in determining the cytotoxic effect of the hybrids tested in the RKO and Hep3B2.1-7 cell lines. These findings might indicate that the levels of NO released could be insufficient to induce cell death.

## 4. Materials and Methods

### 4.1. Synthesis and Characterization

#### 4.1.1. General

Commercial sodium azide (NaN_3_) and sodium ascorbate were purchased from Sigma-Aldrich (St. Louis, MO, USA); copper sulphate (CuSO_4_.5H_2_O) was purchased from Fluka (Buchs, Switzerland). All the chemicals were used without further purification. The reactions were monitored by TLC on pre-coated silica gel F254 plates (thickness 0.25 mm, Merck, Darmstadt, Germany) developed with phosphomolybdic acid solution. Flash column chromatography was performed on silica gel (60 Å, 230–400 mesh, Merck, Darmstadt, Germany). Microwave irradiation was performed by using a Biotage Initiator apparatus (Biotage AB, Uppsala, Sweden). NMR spectra were recorded with a Varian Mercury 400 MHz instrument (Varian, Palo Alto, CA, USA) in the stated solvent. MS analyses were performed on a triple quadrupole mass spectrometer TSQ Quantum Access Max and an electrospray ionization source detector.

The synthesis of alkynes **3** and **4** was performed following the procedure previously reported by our group [38,39]. NMR and MS analyses for both compounds were identical to those reported in the literature [38,39]. ^1^H NMR and ^13^C NMR spectra of compounds **2** and hybrids dU-t-NO and dAdo-t-NO were reported in Supplementary Materials. 

#### 4.1.2. Synthesis of N-(5-Azidopentyl)-4-Nitro-3-(trifluoromethyl)aniline (**2**)

NaN_3_ (195 mg, 3.0 mmol) was added in one portion to a stirred solution of compound 1 (1.0 mmol, 355 mg) in dry DMF (10 mL). The reaction mixture was stirred in a sealed tube at 60 °C for 4 h, then poured into water (10 mL) and extracted twice with Et_2_O (12 mL). The combined organic layers were dried by using anhydrous Na_2_SO_4_, filtered, and evaporated under reduced pressure to produce the azide **2** as a light yellow amorphous solid (290 mg, 91%) which was used without further purification. ^1^H NMR (400 MHz, CDCl_3_) δ 8.01 (d, *J* = 9.0 Hz, 1H), 6.87 (d, *J* = 2.7 Hz, 1H), 6.64 (dd, J = 9.1, 2.7 Hz, 1H), 4.65 (bs, NH), 3.32 (t, *J* = 6.6 Hz, 2H), 3.24 (td, *J* = 7.1, 5.5 Hz, 2H), 1.78–1.60 (m, 4H), 1.57–1.44 (m, 2H). ^13^C NMR (101 MHz, CDCl_3_) δ. 151.9, 136.6, 129.3, 126.8 (q, *J*_CF_ = 32.5 Hz), 122.4 (q, *J*_CF_ = 280.0 Hz) 112.6, 111.3 (q, *J*_CF_ = 7.0 Hz), 51.3, 43.4, 28.7, 28.6, 24.3. MS (ES+): *m*/*z* calcd for C_12_H_14_F_3_N_5_O_2_ + H^+^ 318.28 [M + H]^+^ found 318.06.

#### 4.1.3. General Procedure for the Click Reaction

Sodium ascorbate (0.015 mmol), CuSO_4_·5H_2_O (0.005 mmol), and azide 2 (0.10 mmol) were added to a solution of the appropriate alkyne 3 or 4 (0.10 mmol) in 1.5 mL of a 1:1:1.5 mixture of H_2_O/t-BuOH/THF (*v*/*v*). The resulting mixture was premixed for 30 s and then heated in a sealed glass tube in a Biotage Initiator microwave apparatus at 80 °C for 30 min. After cooling at room temperature, the solvent was removed in vacuo and the crude material was partitioned between CH_2_Cl_2_ (2 × 5 mL) and H_2_O (5 mL). The combined organic layers were dried by using anhydrous Na_2_SO_4_, filtered, and evaporated under reduced pressure to produce a crude compound which was purified by flash chromatography.

Hybrid dU-t-NO flash chromatography: Cyclohexane/EtOAc 1:1; 68% yield. ^1^H NMR (400 MHz, CD_3_OD) δ 8.24 (s, 1H,), 8.01 (d, *J* = 9.0 Hz, 1H), 7.78 (s, 1H), 6.95 (d, *J* = 2.5 Hz, 1 H), 6.72 (dd, *J* = 9.2, 2.6 Hz, 1H), 6.23 (t, *J* = 6.6 Hz, 1H), 4.44–4.34 (m, 3H), 3.96–3.83 (m, 1H), 3.88–3.67 (m, 2H), 3.18 (t, *J* = 7.0 Hz, 2H), 2.71 (t, *J* = 7.5 Hz, 2H), 2.40 (t, *J* = 6.9 Hz, 2H,), 2.34–2.15 (m, 2H), 1.94 (p, *J* = 7.1 Hz, 2H), 1.81 (p, *J* = 7.6 Hz, 2H), 1.67 (p, *J* = 7.0 Hz, 2H), 1.58 (p, *J* = 6.9 Hz, 2H) 1.46–1.30 (m, 2H). ^13^C NMR (101 MHz, CD_3_OD) δ 164.7, 154.5, 151.2, 149.0, 144.3, 136.0, 130.6, 127.3 (q, *J*_CF_ = 32.5 Hz), 124.0 (q, *J*_CF_ = 280.0 Hz), 123.3, 120.0, 112.6, 112.2, 101.2, 94.7, 89.1, 86.8, 73.0, 72.0, 62.5, 51.1, 43.7, 41.7, 30.9, 29.5, 29.0, 28.9, 25.8, 24.9, 19.9. MS (ES+): *m*/*z* calcd for C_29_H_34_F_3_N_7_O_7_ + H^+^ 649.25 [M + H]^+^ found 650.12.

Hybrid dAdo-t-NO: flash chromatography: Cyclohexane/EtOAc 1:1; 65% yield. ^1^H NMR (400 MHz, CD_3_OD) δ 8.14 (s, 1H), 7.98 (d, *J* = 9.2 Hz, 1H), 7.79 (s, 1H), 6.94 (d, *J* = 2.6 Hz, 1H), 6.69 (dd, *J* = 9.2, 2.6 Hz, 1H), 6.59 (dd, *J* = 9.0, 5.9 Hz, 1H), 4.63 (dt, *J* = 5.5, 1.8 Hz, 1H), 4.39 (t, *J* = 7.0 Hz, 2H), 4.11 (td, *J* = 2.9, 1.6 Hz, 1H), 3.88 (dd, *J* = 12.5, 2.6 Hz, 1H), 3.74 (dd, *J* = 12.5, 3.1 Hz, 1H), 3.17 (t, *J* = 7.0 Hz, 2H), 3.03 (ddd, *J* = 13.3, 9.1, 5.6 Hz, 1H), 2.77 (t, *J* = 7.4 Hz, 2H), 2.63 (t, *J* = 6.9 Hz, 3H), 2.28 (ddd, *J* = 13.3, 6.0, 1.7 Hz, 1H), 2.00–1.92 (m, 3H), 1.91–1.84 (m, 2H), 1.77–1.69 (m, 2H), 1.65 (dt, *J* = 14.9, 7.2 Hz, 3H), 1.45–1.33 (m, 2H). ^13^C NMR (101 MHz, CD_3_OD) δ 157.2, 154.5, 153.9, 149.5, 148.7, 136.1, 135.9, 130.5, 127.3 (q, *J*_CF_ = 32.5 Hz), 124.0 (q, *J*_CF_ = 280 Hz), 123.3, 120.6, 112.6, 112.1, 100.0, 90.5, 88.3, 73.8, 70.6, 64.3, 51.1, 43.7, 40.3, 30.9, 29.7, 29.1, 28.4, 25.7, 24.9, 19.7.MS (ES+): *m*/*z* calcd for C_30_H_35_F_3_N_10_O_5_ + H^+^ 672.27 [M + H]^+^ found 673.11.

#### 4.1.4. Chemical Stability of Hybrids

A DMSO mother solution of each hybrid at 20 mM concentration was prepared. The solution was diluted in complete cell culture medium (DMEM) to a final concentration of 50 or 100 μM. The solutions were kept at 37 °C in the dark. A time course at 3, 6, and 14 h was considered. Chromatograms at the relevant detection wavelength of 420 nm for all hybrids were extracted from PDA data (600–200 nm). The experiment was performed in duplicate.

HPLC-UV-MS analyses were performed on an Agilent 1260 Infinity HPLC equipped with a triple quadrupole mass spectrometer TSQ Quantum Access Max and electrospray ionization source detector. Samples of 0.5 mL were used as sources for the automated injection. LC-MS grade methanol was purchased from Sigma-Aldrich (Merck, Italy) in the highest available purity and was used without any further purification. Ultrapure water (uH_2_O) was produced using a Sartorius Arium Pro^®^ system (Goettingen, Germany). The chromatographic separation was performed on a reverse phase Zorbax C8 column 4.6 × 150 mm, 5 μm (Agilent Technologies, CA, USA), at a flow rate of 0.5 mL/min. Linear gradient: mobile phases A (NH_4_OAc 5 mM and HCOOH 0.1% in uH_2_O *v*/*v*) and B (MeOH) from 50:50 to 5:95. MS analyses were performed in full scan mode: scan range 250–1500, positive ionization (ES+).

### 4.2. Photochemistry

#### 4.2.1. Photochemical Quantum Yield Measurements

Photochemical quantum yield determinations were accomplished using the 2nd harmonics (355 nm) of a Q-switched Continuum Surelite II Nd:YAG laser delivering pulses of 5 ns duration at a maximum repetition frequency of 10 Hz.

#### 4.2.2. Irradiation Apparatus for In Vitro Experiments

The custom-made irradiation apparatus used in experiments on cells comprised an aluminum frame support bearing at the base of a 100 mm × 80 mm matrix of 48 LEDs, derived from a SMD5050 UV LED strip and powered by a 24 V dc power supply Mean Well model HLG-100H-24B. Each UV LED was capable of generating up to 10 mW of optical radiant power at a peak wavelength of 380 nm. The frame could accommodate standard cell culture plates positioned at an adjustable height to regulate the optical power density at the desired value.

**Figure 11:**
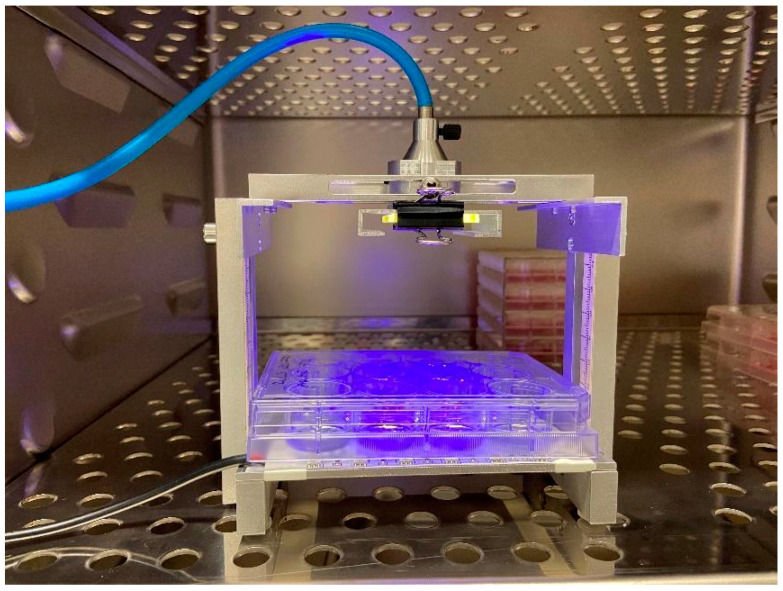
Custom-made irradiation apparatus for in vitro experiments.

As an alternative, the device was provided with a source radiating from top to bottom realized by coupling one of the six LEDs of an Intelligent LED strip Solutions model ILS-XC06-S410-SD111 to an optical fiber mounting a collimating lens at the exit end fixed to the support frame. The single LED source was horizontally and vertically adjustable to yield uniform illumination over about a 5 cm diameter spot. The peak wavelength of the single LED was at 420 nm and the power density was kept in the range of 0.5–1.5 m W/cm^2^ over the cell plane. A colored glass cut-off Schott GG420 filter was used to reshape the LED spectral profile in order to eliminate wavelengths below 410 nm (Figure 11).

#### 4.2.3. Griess Assay

The photogeneration of NO was checked by evaluating the amount of nitrite formed upon irradiation of the hybrids for 40 min with the 420 nm LED source using the Griess Reagent System (Promega, Madison, WI, USA), following the manufacturer’s instructions.

### 4.3. Biological Evaluation

#### 4.3.1. Cell Lines

The evaluation of the biological effects of the three hybrids was performed on the human RKO colon carcinoma cell line and on the human hepatocarcinoma cell line Hep 3B2.1-7, both purchased from the American Type Culture Collection (ATCC, Manassas, VA, USA). RKO and Hep 3B2.1-7 cells were cultured in Dulbecco’s Modified Eagle Medium (DMEM; Corning, Glendale, AZ, USA) supplemented with 10% fetal bovine serum (FBS; Gibco, Grand Island, NY, USA), 2 mM L-glutamine, 100 U/mL penicillin, and 100 mg/mL streptomycin (L-Glutamine–Penicillin–Streptomycin solution from Sigma-Aldrich, St. Louis, MO, USA). All the cell lines were maintained at 37 °C in an atmosphere of 5% CO_2_ and 90% relative humidity.

#### 4.3.2. Evaluation of the Cytotoxic and Cytostatic Effects of the Hybrids

For the evaluation of the biological effects of the hybrids, the molecules were all reconstituted in DMSO, whose final concentration never exceeded 0.1%.

In a first set of experiments, RKO and Hep 3B2.1-7 cell lines were treated for 36 or 72 h with different concentrations of dAdo-S-NO, dAdo-t-NO, and dU-t-NO (range: 10–100 μM) and maintained in the dark. After 36 h of treatment, cell cycle analysis was performed by flow cytometry. For this purpose, 5-bromodeoxyuridine (BrdU) was incorporated into newly synthesized DNA for 1.5 h at 37 °C; the incorporated BrdU was then detected using a primary anti-BrdU antibody (BioLegend, San Diego, CA, USA) and the complex was revealed using a FITC conjugated secondary antibody (Cytek Biosciences, Fremont, CA, USA). Finally, cells were stained with propidium iodide (PI, 50 μg/mL, Sigma-Aldrich) and analyzed using a FACSCalibur flow cytometer (BD Biosciences, San Josè, CA, USA).

The hybrid effects on RKO and Hep 3B2.1-7 cell viability and proliferation were evaluated after 72 h of treatment by counting cells with Trypan blue and by an MTT colorimetric assay (Roche Diagnostics Corporation, Indianapolis, IN, USA), following the manufacturer’s instructions. MTT assay quantification was performed using a TECAN Infinite^®^ M Plex microplate reader (Tecan Trading AG, Männedorf, Switzerland, CH). The cytotoxicity of the hybrids was also confirmed by the analysis of the percentage of apoptosis by flow cytometry. For this purpose, cells were stained with annexin V-FITC and PI (Beckman Coulter Inc., Brea, CA, USA). Cell cycle and apoptosis data analysis was performed using the FloJo Software (Tree Star, Ashland, OR, USA).

For a comparative evaluation of the hybrids’ IC_50_ values, the RKO cell line was also treated with a scalar dose of Paclitaxel (range: 1–500 nM), used as a reference drug, following the method previously described [43]. In particular, cells were incubated with Paclitaxel for 4 h and then treatments were removed and replaced with fresh complete medium; after a further 72 h, viable cell number and apoptosis were evaluated as described above.

#### 4.3.3. Evaluation of the Effects of Irradiation on dAdo-t-NO and dU-t-NO Effectiveness

A second set of experiments was performed to evaluate the contribution of NO release on cell viability. In this respect, RKO cells were treated for 72 h with 50 and 100 μM of dAdo-t-NO or dU-t-NO hybrids, while Hep3B2.1-7 cells were treated with 50 and 100 μM of the dAdo-t-NO hybrid. After 12 h of treatment, both cell lines were irradiated for 40 min by using the custom-made apparatus described above (Figure 11). Irradiation was performed at 37 °C in the presence of 5% CO_2_ and 90% relative humidity. After irradiation, cells were incubated until 72 h after the initial treatment, then they were analyzed to evaluate dAdo-t-NO or dU-t-NO cytotoxicity using a Trypan blue dye exclusion count and apoptosis analysis, as described above. Parallel to these experiments, cells were also subjected to the same treatments in the dark, as a control.

#### 4.3.4. Statistical Analysis

For the evaluation of the hybrids’ biological effects, data results, obtained from at least three independent experiments, were analyzed by one-way ANOVA followed by the Bonferroni post-hoc test (for multiple corrections) using GraphPad Prism software, version 8.4.2 (GraphPad Software, San Diego, CA, USA). Results were expressed as mean ± standard deviation (SD) of replicate experiments. Statistical significance was defined as almost *p* < 0.05.

IC_50_ values were calculated using GraphPad Prism software, version 8.4.2 (GraphPad Software).

## 5. Conclusions

In this work, we reported on the biological evaluation of the 2′-deoxyadenosine derivatives dAdo-S-NO and dAdo-t-NO, as well as the newly synthesized 2′-deoxyuridine hybrid dU-t-NO, prepared by click chemistry and endowed with a NO PD unit. The light-triggered release of NO has been demonstrated by spectroscopic and photochemical studies. All hybrids showed the effective release of NO in the μM range. The hybrids tested in the dark in both RKO colon- and Hep3B2.1–7 hepatocarcinoma cell lines showed interesting cytostatic and cytotoxic effects with IC_50_ values in the range of 10.39–61.66 μM with a slight cytoselectivity towards the RKO colon carcinoma cell line. Moreover, all hybrids displayed a more marked pro-apoptotic effect in RKO cell line with respect to Hep 3B2.1-7. The SAR study in the dark suggested that 2′-deoxyadenosine-based hybrids (dAdo-S-NO and dAdo-t-NO) can be considered more interesting, showing higher cytotoxicity and selectivity than the 2′-deoxyuridine derivative (dU-t-NO).

Attempts to improve the cytotoxic and antiproliferative effects of dU-t-NO in the RKO cell line and dAdo-t-NO in both RKO and Hep3B2.1-7 cell lines by the light-triggered release of NO failed under the photochemical conditions employed. Indeed, both dU-t-NO and dAdo-t-NO hybrids showed no evidence of the effect of NO release in terms of both cell viability and apoptotic effect in the selected cells upon illumination.

Specific NO donors such as NO PD units able to control NO production and release specifically at the target site have been considered of particular interest due to the intrinsic difficulties in the direct administration of NO [28]. In particular, the PD unit 4-nitro-3-(trifluoromethyl)aniline was chosen not only because of its synthetic versatility but also because it has been reported as being non-toxic to cells before and after the release of NO [34]. Indeed, the mechanism of NO release leads to phenol-derivative photoproducts (Figure 1A) that, according to similar compounds, are potential antioxidants. This represents a possible advantage with respect to other NO photo-generators in terms of the photoproduct’s cytotoxicity [44,45]. However, the mechanism of action of 4-nitro-3-(trifluoromethyl)aniline conjugated in light conditions has not been fully elucidated yet, as also reported in the literature for other hybrid compounds [46]. Therefore, the hybrids herein reported, also in consideration of the intrinsic cytotoxicity displayed towards colon- and hepatocarcinoma cell lines, should be further investigated in order to clarify the missed effectiveness of NO. In particular, a lack of bioavailability of dU-t-NO and dAdo-t-NO hybrids might, at least in part, account for the unsuccessful results. Hence, further studies focused on hybrid nano-based delivery systems will be planned.

## Figures and Tables

**Table 1:** IC_50_ values ± SEM (standard error of the mean) of each hybrid and of Paclitaxel, reported for the two studied cell lines, RKO and Hep 3B2.1-7.

	RKO	Hep 3B2.1-7
**Compound**	IC_50_ (μM)	IC_50_ (μM)
**dAdo-S-NO**	10.39 ± 0.13	17.64 ± 2.19
**dAdo-t-NO**	27.61 ± 1.22	44.46 ± 3.45
**dU-t-NO**	51.74 ± 2.69	61.66 ± 5.29
**Paclitaxel**	5.01 × 10^−3^ ± 0.74 × 10^−3^	0.41 ± 0.12

## Data Availability

Data are contained within the article and Supplementary Materials.

## Supplementary Materials

The following supporting information can be downloaded at: https://www.mdpi.com/article/10.3390/molecules29143383/s1, Figure S1: NMR spectra of (5-azido-pentyl)-(4-nitro-trifluoromethyl-phenyl) amine (2); Figure S2: NMR spectra of hybrid dU-t-NO; Figure S3: NMR spectra of hybrid dAdo-t-NO.
