# Supplementary material for: Evaluation of Anticancer Activity of Nucleoside–Nitric Oxide Photo-Donor Hybrids
Source: Molecules. 2024 Jul 18;29(14):3383. doi: 10.3390/molecules29143383 (PMC11279448; doi:10.3390/molecules29143383)
Supplement: Supplementary file 1 [file molecules-29-03383-s001.zip › molecules-3077685-supplementary.pdf]

Evaluation of anticancer activity of nucleoside-nitric oxide photodonor hybrids

Elena Marchesi[1,†], Elisabetta Melloni[2,,†], Fabio Casciano[2,] , Elena Pozza[3], Roberto Argazzi,[1,4,] Carmela De Risi[1], Lorenzo Preti[5], Daniela Perrone[5,*] and Maria Luisa Navacchia[4,*]

1 *Department of Chemical, Pharmaceutical and Agricultural Sciences, University of Ferrara, 4412 Ferrara, Italy*
2 *Department of Translational Medicine and LTTA Centre, University of Ferrara, 44121 Ferrara, Italy*
3 *Department of Translational Medicine, University of Ferrara, 44121 Ferrara, Italy*
4 *Institute for Organic Synthesis and Photoreactivity (ISOF), National Research Council of Italy (CNR), I-40129 Bologna, Italy*
5 *Department of Environmental and Prevention Sciences, University of Ferrara, 44121 Ferrara, Italy*

* Correspondence: marialuisa.navacchia@isof.cnr.it (M.L.N.); prd@unife.it (D.P.)
† These authors equally contributed to the work

Figure S1: NMR spectra of *N*-(5-azidopentyl)-4-nitro-3-(trifluoromethyl)aniline (2)

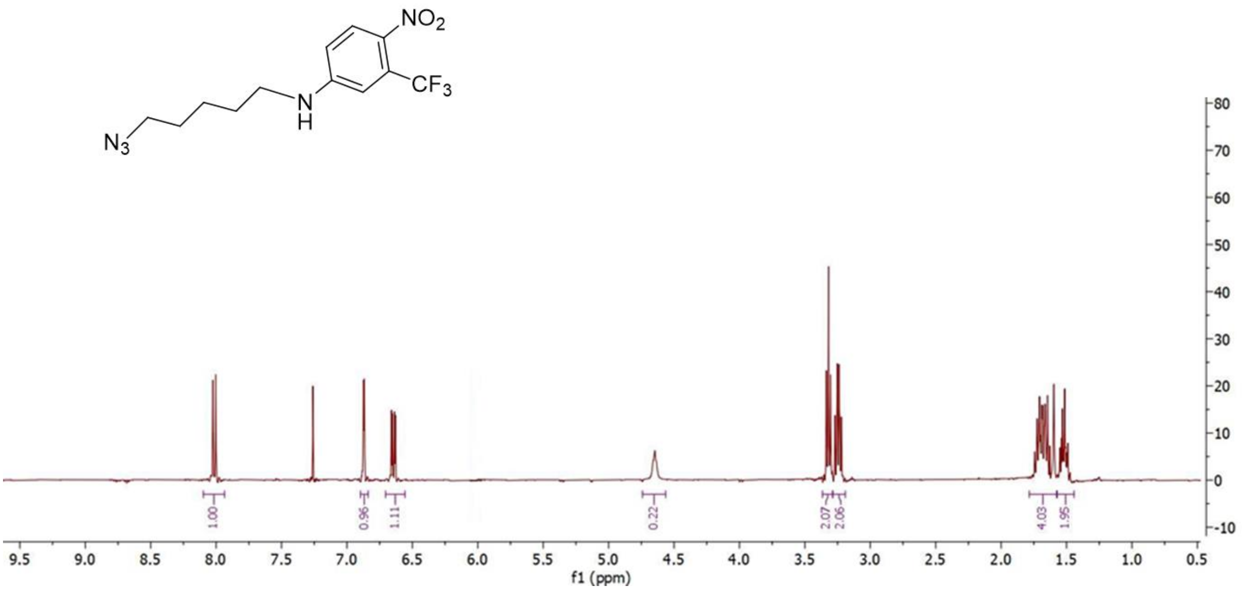

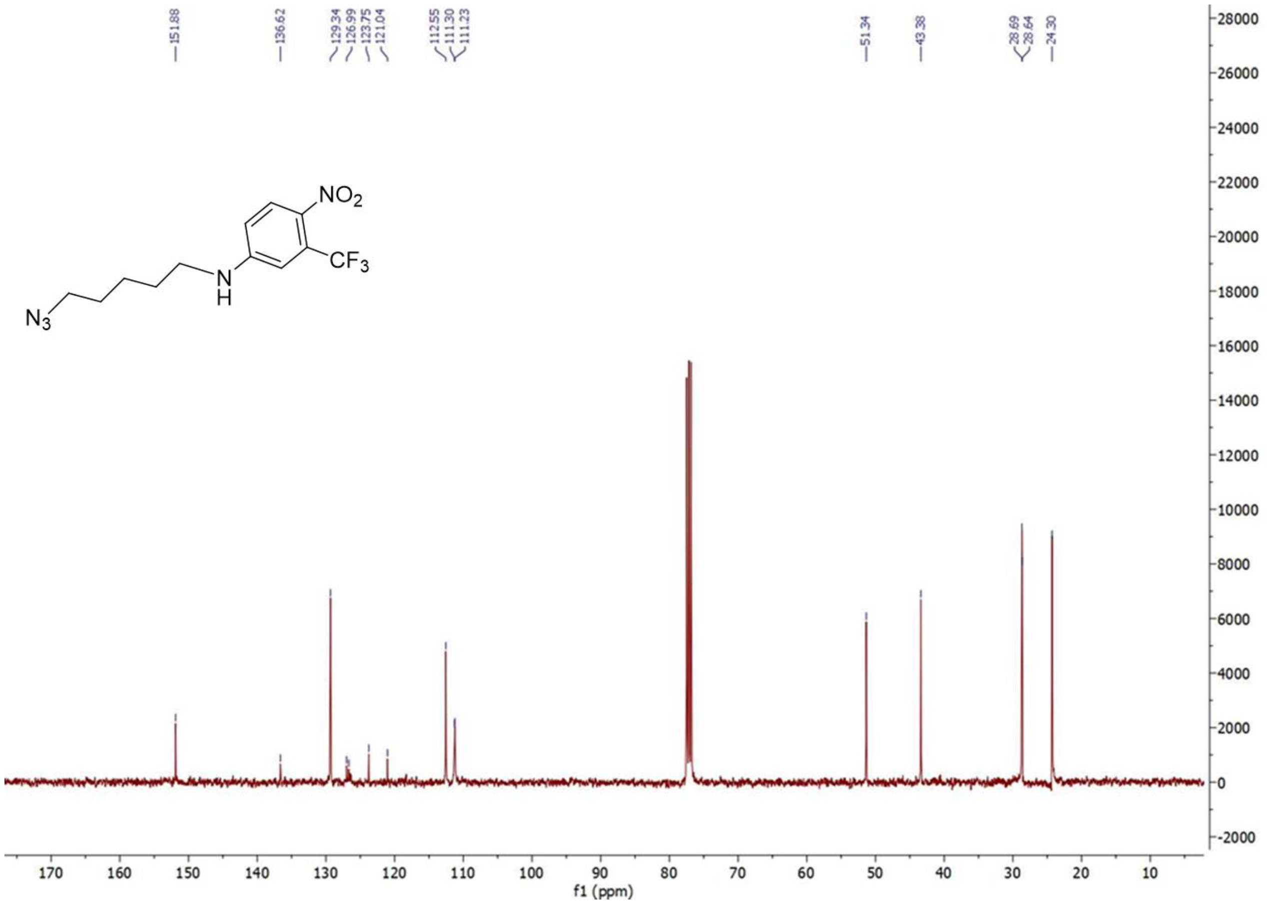

Figure S2: NMR spectra of hybrid dU-t-NO

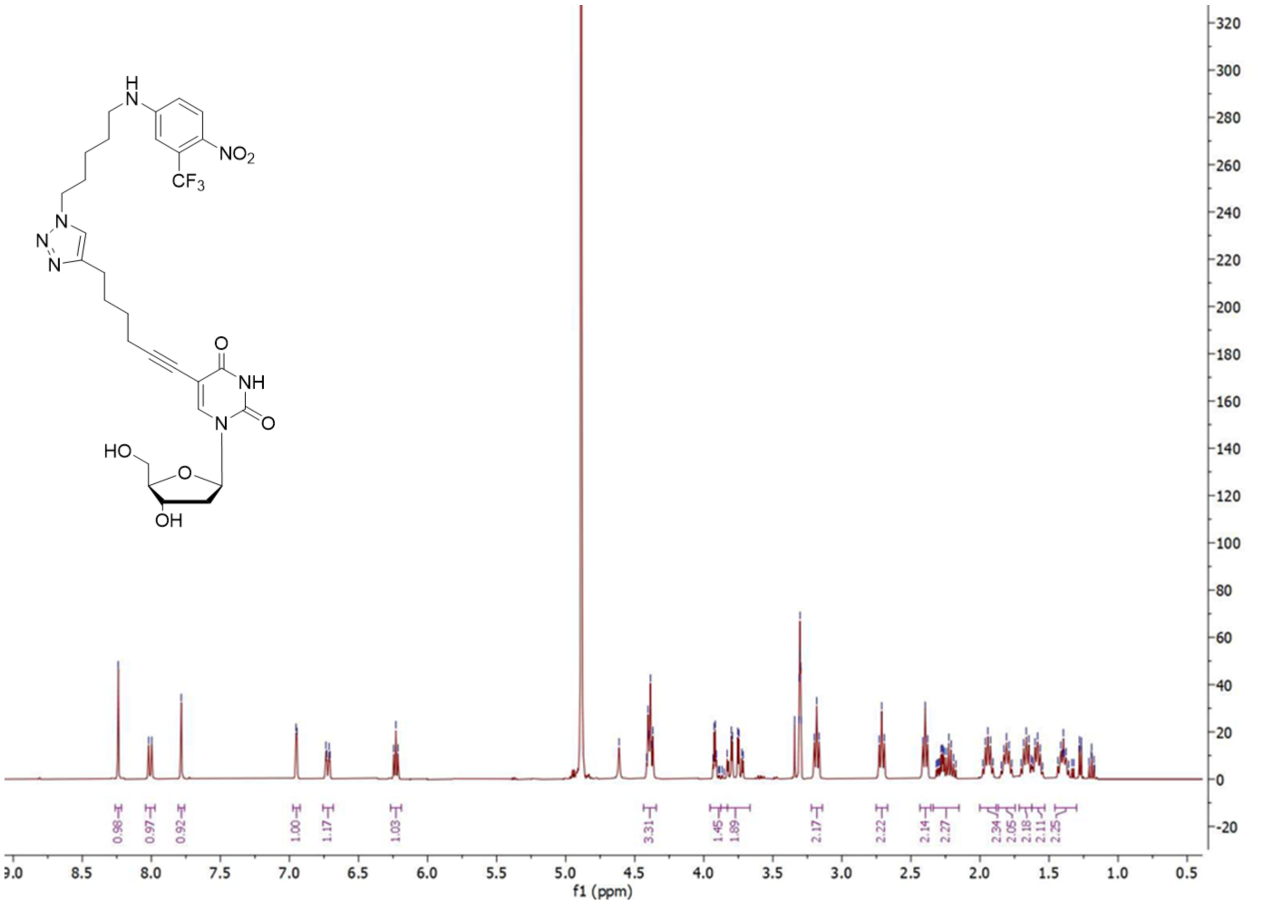

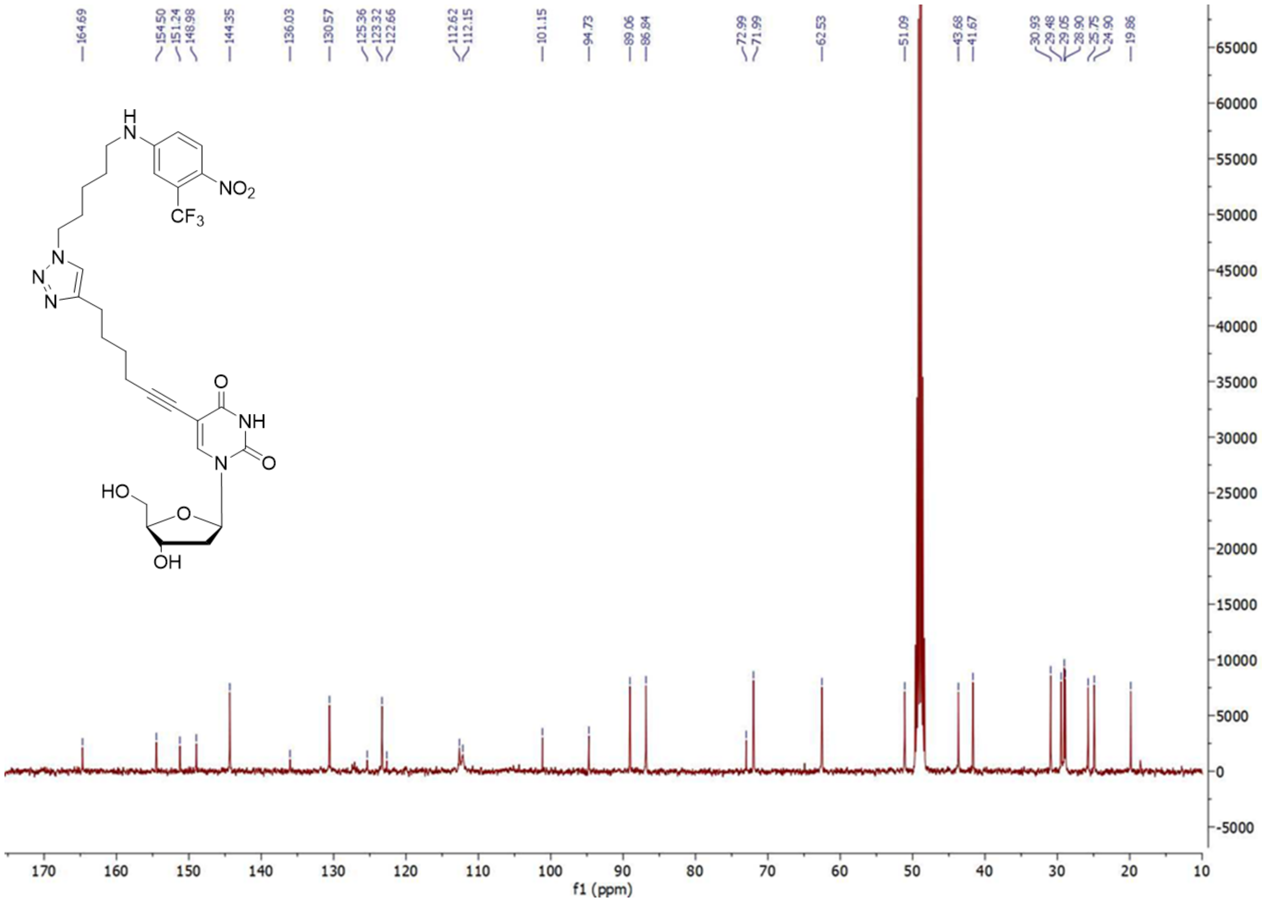

Figure S3: NMR spectra of hybrid dAdo-t-NO

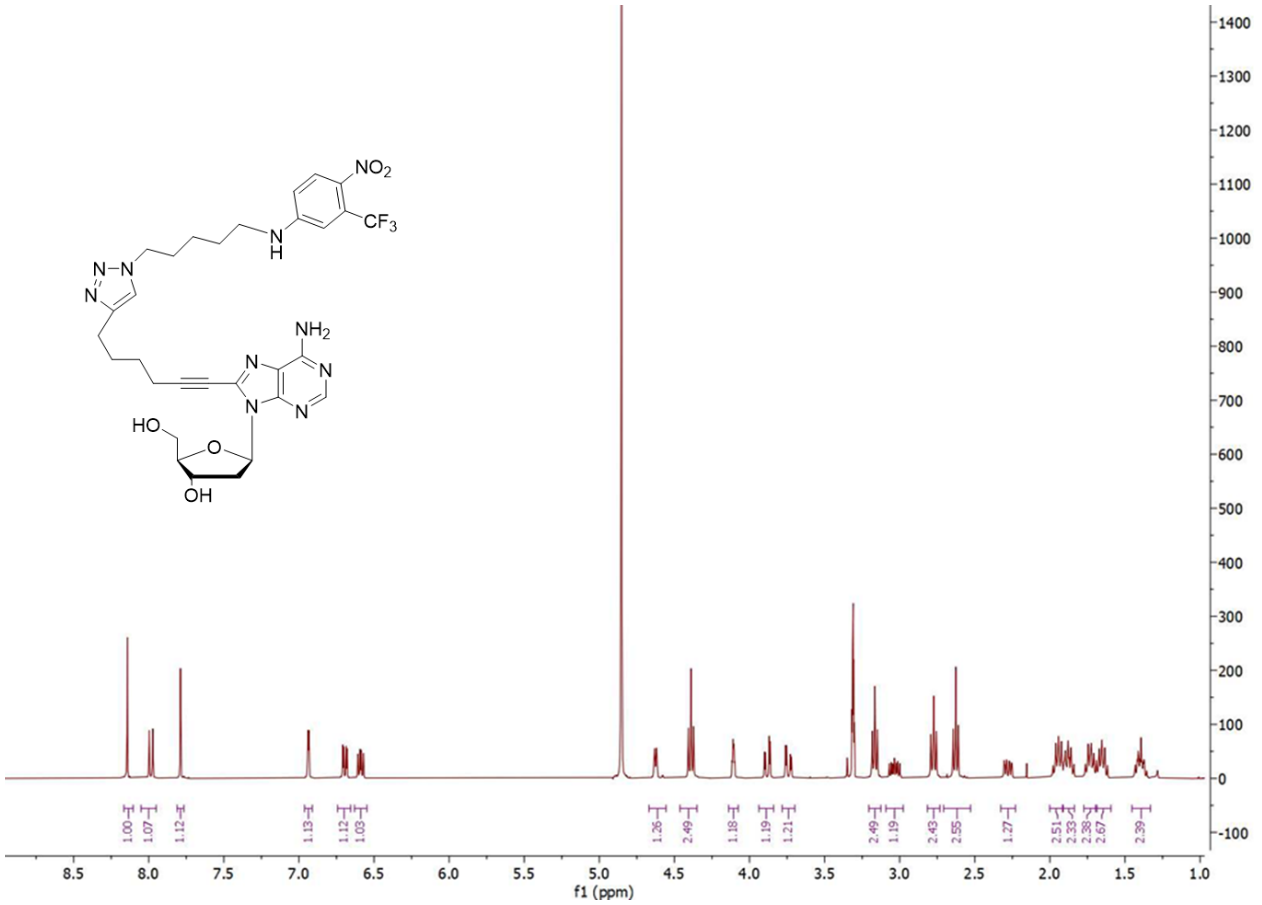

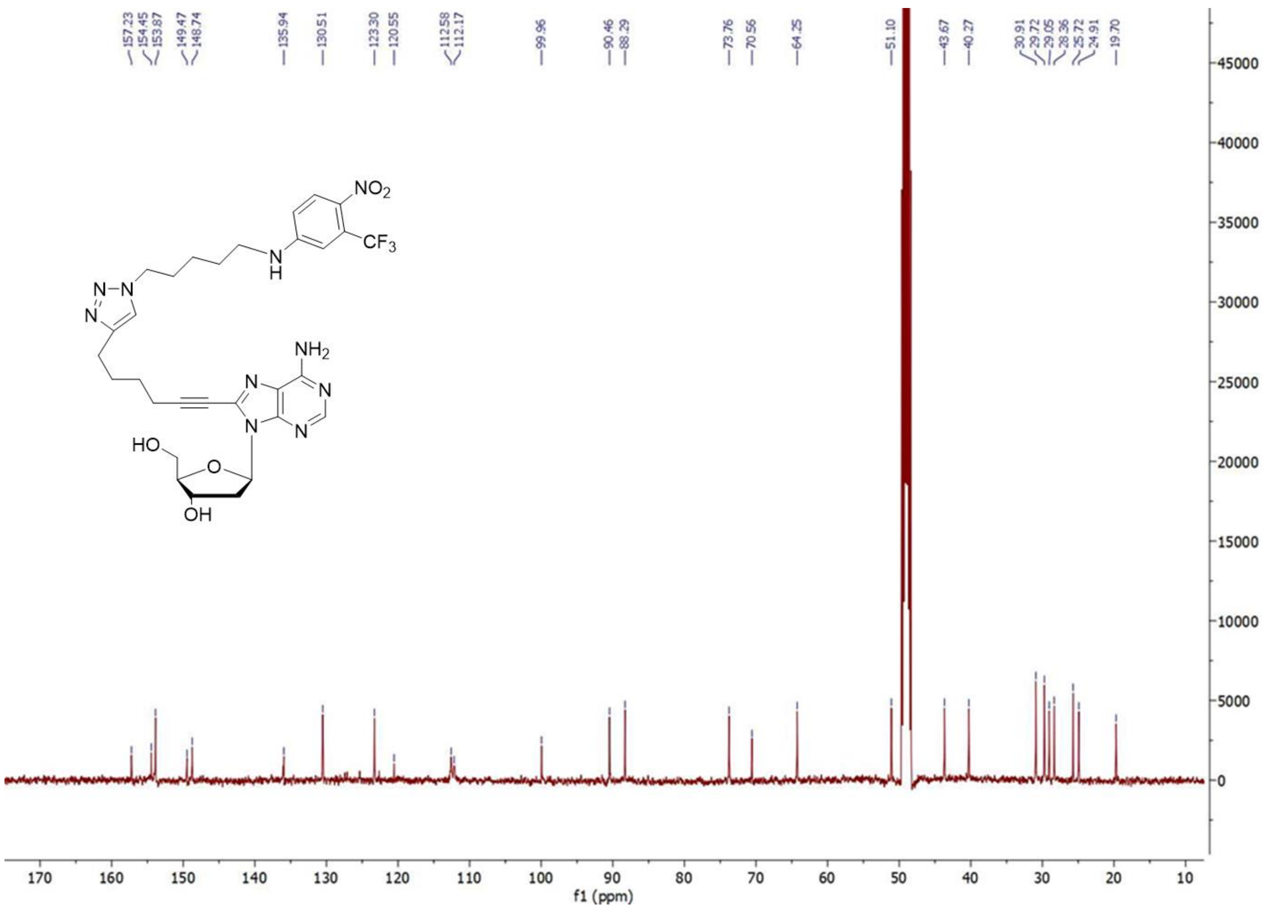

157.23
154.45
153.87
149.47
148.74
135.94
130.51
123.30
120.55
112.58
112.17
99.96
90.46
88.29
73.76
70.56
64.25
51.10
43.67
40.27
30.91
29.72
29.05
28.36
25.72
24.91
19.70
NO2
CF3
N
H
N
N
N
NH2
N
N
N
N
HO
O
OH
45000
40000
35000
30000
25000
20000
15000
10000
5000
0
170 160 150 140 130 120 110 100 90 80 70 60 50 40 30 20 10
f1 (ppm)
